# The Expression Pattern of Ferroptosis-Related Genes in Colon Adenocarcinoma: Highly Correlated to Tumor Microenvironment Characteristics

**DOI:** 10.3389/fgene.2022.837941

**Published:** 2022-03-08

**Authors:** Jie Liu, Hui Li, Shen Zhao, Rongbo Lin, Jiaqing Yu, Nanfeng Fan

**Affiliations:** Department of Gastrointestinal Medical Oncology, Fujian Cancer Hospital, Fujian Medical University Cancer Hospital, Fuzhou, China

**Keywords:** prognosis, immune status, tumor microenvironment, ferroptosis, colon cancer

## Abstract

In the latest literatures, ferroptosis caused by T cells in cancerous cells provided new insights of improving curative effect of the PD-1/PD-L1 antibody. The microenvironment on which tumor cells develop and survive was also emphasized as its crucial role in tumor occurrence, development, metastasis and immune escape. Thus, the interaction of ferroptosis related genes and tumor microenvironment (TME) was urgently be detected in a comprehensive perspective. We comprehensively evaluated the transcriptional feature of ferroptosis related genes in colon adenocarcinoma (COAD), and systematically associated these ferroptosis subtypes with DNA damage repair (DDR) and TME characteristics. We found two unique patterns of ferroptosis characterized by distinct biological pathways activation. We also demonstrated that FRG score constructed based on ferroptosis subtypes has a significant correlation with prognosis of colon cancer and could act as an independent prognostic biomarker for predicting patients’ survival. The higher immune infiltrating level, immune functional pathways activation was observed in the high FRG score group. Furthermore, these results were verified by an independent external GEO cohort. This work revealed ferroptosis was highly associated with TME complexity and diversity. A novel ferroptosis subtypes related gene scoring system can be used for prognostic prediction in COAD. Targeting ferroptosis may be a therapeutic alternative for COAD.

## Introduction

Ferroptosis is a programmed cell death characterized by an iron-dependent oxidative alteration of phospholipid membranes (Conrad et al., 2016). A preliminary analysis of this route established that depletion of cysteine, which results in the exhaustion of the intracellular glutathione (reduced) (GSH) pool, initiates this type of cell death (Wang, et al., 2019) explicitly. The GSH requirement for ferroptosis protection is connected to the optimum functioning of the enzyme glutathione peroxidase 4 (GPX4). A selenoprotein is necessary to effectively reduce peroxidized phospholipids and suppress arachidonic acid (AA)-metabolizing enzyme activity. This may contribute to the phospholipid peroxidation process (Friedmann Angeli et al., 2014); (Yang et al., 2014); (Ingold et al., 2018); (Kagan et al., 2017). Since then, it has become clear that a complex interaction between lipid, iron, and cysteine metabolism is critical for this cell death process. Ferroptosis has been implicated in the development and progression of tumors by activating various regulatory sites in signaling pathways, promoting tumor cell death (Friedmann Angeli et al., 2019) (Tang R. et al., 2020). Thus, elucidating the ferroptosis process and the associated mechanisms that regulate tumor formation may yield novel therapeutic strategies for malignancies (Wu et al., 2020); (Hassannia et al., 2019).

Immunotherapy, represented by the immune checkpoint blockade (ICB, PD-1/L1, and CTLA-4), has shown significant clinical success in a limited proportion of patients with long-lasting responses. Unfortunately, most patients show slight or no clinical improvement, far from meeting a clinical requirement (Topalian et al., 2012). Historically, tumor growth was thought to be a multistep process involving primarily epigenetic and genetic changes inside tumor cells. However, many reported studies have demonstrated that the microenvironment on which tumor cells develop and survive also plays a critical role in the progression of the tumor. In cancer, there is a complex microenvironment made up of macrophages and resident fibroblasts (CAF; cancer-associated fibroblasts) as well as immune cells infiltrating the tumor (myeloid cells and lymphocytes) cells derived from the bone marrow (BMDCs), and secreted factors like cytokines. TAMs, TANs, dendritic cells, myeloid-derived suppressor cells (MDSCs), and Tie2-expressing monocytes were all part of the tumor-associated myeloid cell (TAM) population (TAMCs) (Pitt et al., 2016). With other TME components interactions (directly or indirectly), cancer cells induced several biological behavior changes. Including proliferation and angiogenesis, inhibition of apoptosis, avoidance of hypoxia, and induction of immunological tolerance. As our understanding of the tumor microenvironment’s diversity and complexity increased. Accumulating data demonstrates its crucial involvement in tumor development, immune escape, and immunotherapy response. To improve the efficacy of existing ICBs and to develop novel immunotherapeutic tactics, predicting ICB response based on TME cell infiltration characterization is a critical process (Quail and Joyce, 2013), (Ali et al., 2016). Therefore, the different tumor immune phenotypes could be uncovered through systematically analyzing the TME landscape’s variability and complexity. Additionally, the capability to guide and anticipate immunotherapeutic response would improve.

Several studies have recently demonstrated a unique link between TME-infiltrating immune cells, particularly CD8^+^ T cells, and ferroptosis. Wang et al. found that the ferroptosis-specific lipid peroxidation in tumor cells was induced by immunotherapy-activated CD8^+^ T cells. Ferroptosis contributes to immunotherapy’s anticancer efficiency via interferon-gamma (IFN) released by CD8^+^ T cells (Wang, et al., 2019). The OTUD1 increases transferrin receptor protein-1 (TFRC)-mediated iron transport by deubiquitinating and stabilizing IREB2. Resulting in enhanced ROS production, ferroptosis and potentiates host antitumor immunity (Song et al., 2021). Previous colon adenocarcinoma (COAD) research has, however, been limited to one to several ferroptosis regulators. At the same time, the promising anticancer effect is characterized by many tumor suppressor factors associated in a well-coordinated manner. Widespread recognition of TME cell infiltration characterizations mediated by numerous ferroptosis-related genes will help us better understand Cancer-immune cycle controlled by TME. We integrated the genetic information from multiple colon cancer samples in this study and then identified the ferroptosis subtypes with thoroughly different TME cell-infiltrating features. We also discovered differences in the landscape of DNA damage repair (DDR) and somatic mutations between them. To assess ferroptosis in individual patients, we created a scoring system based on ferroptosis subtype-related genes. Following that, we established a consistent relationship between ferroptosis and immune infiltrating cells.

## Material and Methods

### Acquisition and Preprocessing of Data

We used the Gene-Expression Omnibus (GEO) and Cancer Genome Atlas (TCGA) databanks to obtain clinical annotation and public gene expression data. Colonic cancer cohorts (GSE17536, GSE39582, and TCGA-COAD) were collected for this study. From the University of California Santa Cruz Xena browser, Genomic Data Commons (https://xenabrowser.net/datapages and https://gdc.xenahubs.net accessed online 7 July 2020), the RNA sequencing data (FPKM values) for gene expression were acquired. The normalized matrix files from GEO’s datasets were obtained directly. The FPKM values were converted into TPM (transcripts per kilobase million) values in the next step. The “sva” package’s “ComBat” technique was used to correct batch effects caused by non-biological technical biases. To plot the copy number variation landscape of genes involved in ferroptosis in human DNA, the R package “Rcircos” was used. The somatic mutation data were obtained from the TCGA database, and the data were analyzed through R Bioconductor and R (v.3.6.1) packages.

### Genetic Abnormalities and Highly Significant Tumor Mutational Patterns

The MutSigCV method was used to recognize genes that have undergone substantial mutations (SMGs) (Lawrence et al., 2013). The MutSigCV, in particular, evaluates the significant improvement of nonsilent somatic mutations in a gene through addressing mutational context-specific background mutation rates. The R “maftools” package displayed the mutational landscape of FRGs and SMGs in the TCGA-COAD cohort (Mayakonda et al., 2018). The RCircos R program visualized the copy number difference landscape of 51 differentially expressed FRGs across 23 chromosomal pairs.

### Unsupervised Clustering for Ferroptosis Related Genes With Prognostic Ability

Survival analysis using the software “survminer” and univariate COX regression study was used to discover FRGs with prognostic significance in COAD. Differential analysis between tumor and paired tumor-adjacent tissue, different ferroptosis subtypes were conducted by “limma” package. Fifty-one differentially expressed ferroptosis-related genes were extracted for identifying different ferroptosis subtypes mediated by FRGs. An unsupervised clustering technique was applied to categorize patients into separate ferroptosis subtypes based on the expression of these 51 FRGs. The number of clusters and their stability were established using the consensus clustering technique. We performed the steps mentioned above using the ConsensuClusterPlus program and repeated them 1,000 times to ensure classification stability (Yu et al., 2012).

### Estimation of Immune Infiltrating Cells and Immune Function

The levels of infiltration of various tumor infiltrating immune cells in melanoma were assessed through the R package “CIBERSORT” and the LM22 signature with 1,000 permutations (Yoshihara et al., 2013). ESTIMATE evaluated each melanoma sample’s immune and stromal contents (immune and stromal score) (Newman et al., 2015). The single-sample gene set enrichment analysis (ssGSEA) was used for measuring the relative abundance of 28 immune cell types in the tumor microenvironment (Charoentong et al., 2017). A new study compiled unique feature gene panels for identifying each immune cell type. The relative abundance of each immune cell type was represented by an enrichment score on the MCP counter in ssGSEA analysis and standardized to a unity distribution between 0 and one simultaneously (Becht et al., 2016) and TIMER (Li et al., 2017), CIBERSORT-ABS (Yoshihara et al., 2013), QUANTISEQ (Finotello et al., 2019), Xcell (Aran et al., 2017) and EPIC (Racle et al., 2017). Algorithms were tested to determine the immune responses or cellular components in groups with a high low or a high FRG score. Using a Heatmap, we were able to deduce the variations in immunological response between different algorithms.

### Analysis of the Gene Set Variations

We used GSVA enrichment analysis with the “GSVA” R packages to examine the biological processes that differentiate ferroptosis subtypes. GSVA is a nonparametric and unsupervised technique, frequently used to estimate the variance in pathways and biological process activity in samples from an expression dataset (Hänzelmann et al., 2013). A *p*-value of less than 0.05 was considered statistically significant.

### Differentially Expressed Genes Identification Between Diverse Ferroptosis Subtypes

The prior consensus clustering approach categorized patients into two unique ferroptosis subtypes, and the R package “limma” was used to identify ferroptosis-related differentially expressed genes (DEGs). Data for gene expression was normalized using voom and then passed to the lmFit and eBayes algorithm to generate differential expression statistics. The DEGs were filtered for significance using a log fold change greater than 0.585 and an adjusted *p* value less than 0.05.

### Construction of Principal Component Analysis Ferroptosis Related Genes Related Score

For the DEG prediction analysis, we employed a univariate Cox regression model. The genes with a good prognosis were identified for further research. Following that, we created a ferroptosis-relevant gene signature using principal component analysis (PCA). The signature scores were chosen from the central components 1 and 2. This technique benefits from concentrating the score on the set with the group’s largest block of well-linked (or anticorrelated) genes, whereas down-weighting scores from genes that poorly track with other set members. The FRG score is then determined using a GGI-like mechanism (Sotiriou et al., 2006)1:

FRG Score=∑(PC1i+ PC2i )

Where “I” represents the FRG subtypes-related gene expression.

### Nomogram Construction

In multivariate analysis (*p* < 0.05), the “rms” of the R package was applied to produce the nomogram, which incorporates factors having predictive importance. Predicted and actual survival results were compared using calibration curves. ROC curves with time dependence were also used to assess the nomogram’s accuracy as a predictor.

### Statistical Methods

In this work, R 4.1.0 was used to do statistical analysis. Distance correlation and Spearman analyses were used for obtaining the correlation coefficients between two variables in this study. Statistical significance was determined through Wilcoxon rank-sum test and Student’s t-tests, respectively, for analyzing quantitative data. One-way analysis of variance and Kruskal-Wallis tests were employed for nonparametric and parametric comparisons of more than two groups, respectively (Hazra and Gogtay, 2016). The R package “Survminer” utilized the Cox proportional hazards (v.0.4.6), surv-cutpoint from the “survival” package, and Kaplan-Meier survival analysis to divide samples into law and high FRG score groups. The FRG score model’s prognostic classification performance was calculated through the ROC (receiver operating characteristic) curve, and the “timeROC” program was used to determine the AUC (area under the curve). The hazard ratios (HR) for ferroptosis-related genes and the FRG score were calculated using a univariate Cox regression model. In the multivariate regression model, the patients with comprehensive clinical data were included, and confusing factors were removed. For every *p*-value that fell within the range of 0.05–0.10, it was considered statistically significant. R 4.1.0 software was used for all of the data analysis and visualization.

## Results

### The Transcriptional and Genetic Alterations of Ferroptosis Related Genes in CRC

**FIGURE 1:**
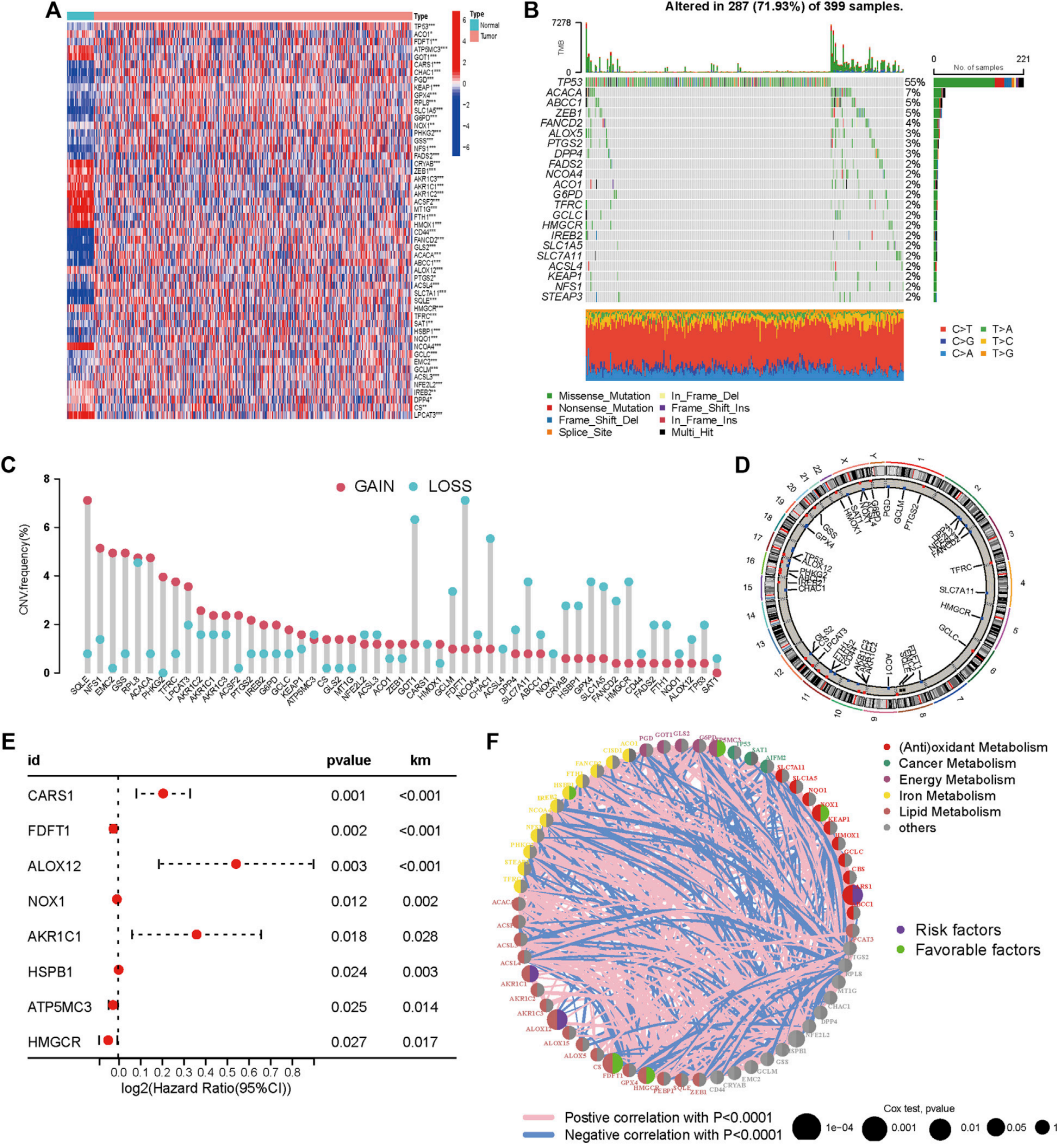
Expression and genetic variation landscapes of m6A regulators with COAD. **(A)** The expression level of ferroptosis related genes in tumor and pericarcinomatous tissue in TCGA-COAD cohort. Blue and red represent low and high expression, respectively. The asterisks are indicative of statistical *p*-value (*p < 0.05; **p < 0.01; ***p < 0.001). **(B)** Mutation frequencies of 22 FRGs (mutation frequency >2%) in 399 patients with COAD from the TCGA cohort. **(C)** Frequencies of CNV gain, loss, and non-CNV among FRGs. **(D)** Locations of CNV alterations in FRGs on 23 chromosomes. **(E)** Univariate Cox regression analysis conducted to estimate the significance of FRGs in prognosis prediction in TCGA-COAD cohort. The horizontal length stands for 95% CI of each gene. **(F)** Association among FRGs within CM. Circle size stands for the impact of every regulator on prognosis prediction. *p* < 0.0001, *p* < 0.001, *p* < 0.01, *p* < 0.05 and *p* < 1, respectively, were obtained from log-rank test. Green and purple dots within the circle indicate protective and risk factors for prognosis, respectively. The lines linking regulators indicate the mutual interactions, with the thickness showing the strength of association between them. Pink and blue colors indicate positive and negative correlation, respectively.

Genes that are involved in ferroptosis were summarized in this study by literature review. Tumors and surrounding nontumorous tissues were found to have significantly different levels of expression for 51/60 (85%) of the ferroptosis-related genes (*p* < 0.05, Figure 1A). Somatic mutations in 22 ferroptosis related genes (FRGs) with more than a 2% mutation rate in the TCGA-COAD cohort were summarized as shown in Figure 1B. FRG mutations were found in 287 of the 399 COAD samples (74.44%) (Figure 1A). TP53, a promising tumor treatment target (Mirgayazova et al., 2019) and ACACA were the two FRGs with the highest mutation rates (55% each), while the mutation rates of the remaining FRGs were all below 5%. We examined the prevalence of CNV alterations in 51 FRGs to see if genetic changes influenced the expression as mentioned earlier variances. The results showed that 51 regulators have a pervasive CNV change. The frequency of CNV amplification was higher than deletion in SQLE, NFS1, EMC2, GSS, ACACA, PHKG2, and TFRC. Furthermore, higher CNV frequency deletions were found in GOT1, GCLM, FDFT1 CHAC1, SLC7A11, SLC7A11, CRYAB, HSBP1, GPX4, SLC1A5, FANCD2, and HMGCR, which indicated that CNV could change gene expression in ferroptosis-related genes.

**FIGURE 2:**
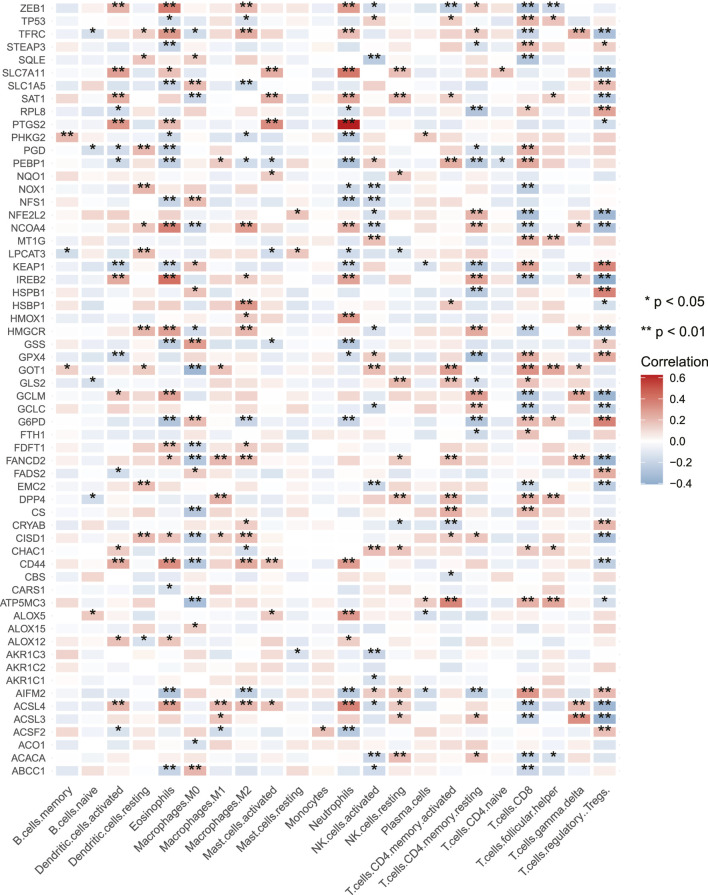
Correlations between the tumor infiltrating immune cells and the expression of FRGs. Blue and red represent low and high expression, respectively. The asterisks are indicative of statistical *p*-value (*p < 0.05; **p < 0.01).

CNV gain-associated FRGs, such as FANCD2, showed decreased mRNA expression. Although CNV can elucidate several assessed variations in FRG expression, CNV is not the only mechanism regulating mRNA expression (Sebestyen et al., 2016). Other variables, such as transcription factors and DNA methylation, can influence gene expression (Koch et al., 2018) (Lambert et al., 2018). In Figure 1D, the locations of CNV alterations in ferroptosis-related genes on chromosomes were shown. In univariate COX analysis, eight prognostic ferroptosis-related DEGs were detected (all FDR< 0.05, Figure 1E). Figure 1F illustrates the network of FRG interactions, prognostic value, and regulator connections in COAD patients. Our findings showed a substantial variation in the genomic characteristics and levels of expression of FRGs among COAD and control samples, suggesting a dormant role for FRGs in COAD oncogenesis. Studies have revealed that ferroptosis can influence tumor immunity by regulating the adaptive immune response (Friedmann Angeli et al., 2019) (Wang, et al., 2019). As a result, we investigated the relationship between tumor-infiltrating immune cells and FRG expression. FRGs have a strong relationship with TIICs, particularly Macrophages M0, Eosinophils, Neutrophils, CD8^+^ T cells, and Tregs (Figure 2).

### Identification of Ferroptosis Subtypes in Colon Adenocarcinoma

**FIGURE 3:**
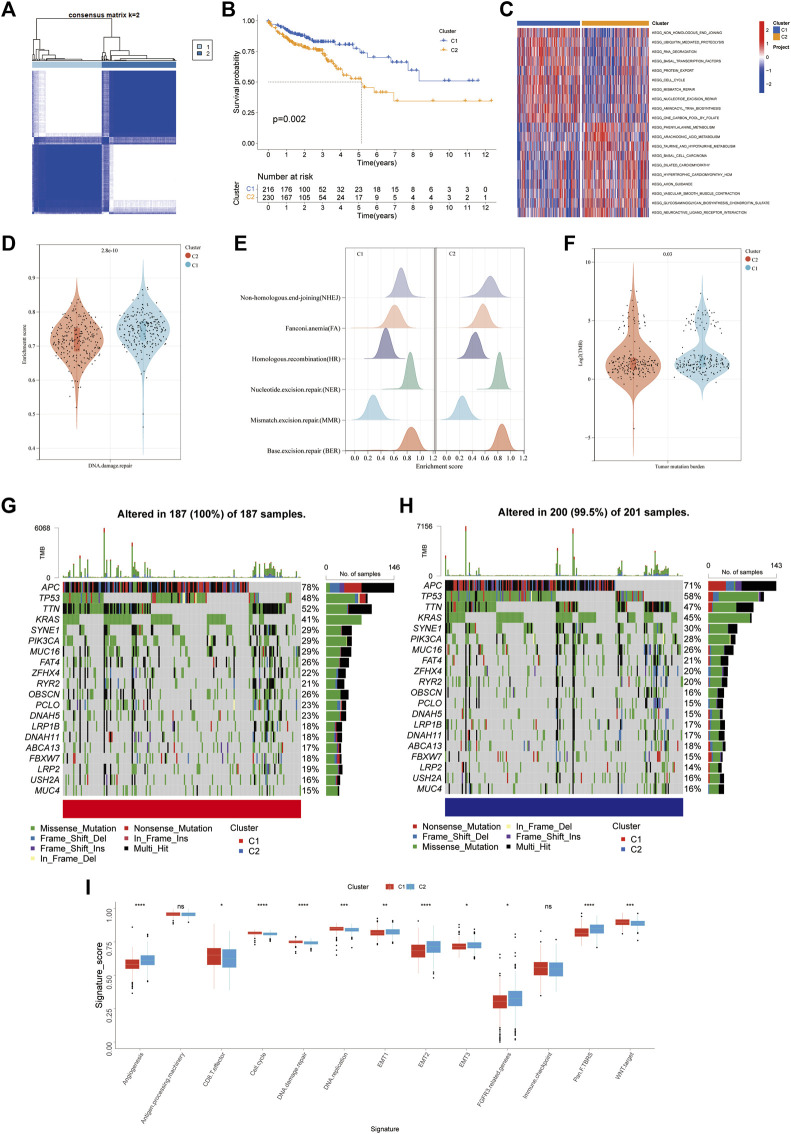
FRGs subtypes, genomic and biological characteristics of two distinct subtypes of samples divided by consistent clustering. **(A)** Consensus matrix heatmap defining two clusters (k = 2) and their correlation area. **(B)** Kaplan-Meier curves for the OS of patients in the ferroptosis cluster 1 and 2. **(C)** GSVA enrichment analysis showing the activation states of biological pathways in distinct ferroptosis subtypes. The heatmap was used to visualize these biological processes, and red represents activated pathways and blue represents inhibited pathways. **(D)** Violin plots for enrichment score of DNA damage repair pathway in cluster 1 and 2. **(E)** Differences in enrichment score of DNA damage repair related pathways including NHEJ, FA, HR, NER, MMR, BER. **(F)** Violin plots for Tumor mutation burden in ferroptosis cluster 1 and 2. g-h. The waterfall plot of tumor somatic mutation established by those with high FRG score **(G)** and low FRG score **(H)** Each column represents individual patients. The upper barplot shows TMB, The number on the right indicates the mutation frequency in each gene. The right barplot showes the proportion of each variant type. **(I)** Differences in stroma-activated and immune-activated pathways among two distinct ferroptosis subtypes (*p < 0.05; **p < 0.01; ***p < 0.001).

To completely understand the expression patterns of FRGs implicated in tumor progression. The patients from the TCGA-COAD cohort were divided into two clusters through a consensus clustering method based on the expression profiles of the 51 differentially expressed FRGs. Our findings indicated that k = 2 seemed to be an ideal selection for categorising the complete population into cluster 1 (C1, n = 216) and cluster 2 (C2, n = 230) subtypes (Figure 3A). The patients classified as C1 had a significantly longer OS than those classified as C2 (log-rank test, *p* =0 .006; Figure 3B) as identified from Kaplan–Meier curves analysis. C1 was strongly enriched in DNA-damaging repair pathways, including nucleotide excision repair, mismatch repair, and non-homologous end joining, as shown by GSVA enrichment analysis.

Meanwhile, we discovered that C1 had activated protein-regulated pathways (KEGG Ubiquitin-mediated proteolysis and KEGG cell cycle) (Figures 3C–E). The GSVA data showed a latent link between DNA damage repair and the expression pattern of FRGs. A total of six signs for DNA damage repair (NHEJ, NER, BER, HR, FA, and MMR) were investigated. Surprisingly, in ferroptosis C1, these pathways were substantially more abundant than in C2 (Figure 3D). Our investigation of the TCGA-COAD cohort’s mutation data revealed a lower TMB in C2 than C1 (Figure 3F). Then, in the TCGA-COAD cohort, we looked at how the distribution of somatic mutations differed across two ferroptosis subtypes. RYR2, ZFHX4, FAT4, MUC16, PIK3CA, SYNE1, KRAS, TTN, TP53, and APC were the top ten mutant genes in both two ferroptosis subtypes (Figures 3G,H). Patients in cluster one showed significantly greater rates of APC, TTN, OBSCN, PCLO and NMAH5 mutations than patients in cluster 2, but the exact opposite was discovered for TP53 and KRAS mutant levels. We also looked at a group of genes linked to Mariathasan et al. identified biological processes (Mariathasan et al., 2018). In cluster 2, EMT markers expression was higher such as EMT3, EMT2, and EMT1, pan-fibroblast TGFβ response characteristics (Pan-F TBRS), angiogenesis characteristics, and FGFR3 associated genes. Cluster one had a considerably higher expression of the CD8 effector (Figure 3I). Previous research found that tumors with an immune excluding phenotype have an abundance of immune cells. On the other hand, instead of invading their parenchyma, these immune cells remained in the stroma surrounding tumor cell nests. In TME, stroma activation was thought to inhibit T-cells (Chen and Mellman, 2017). Stromal activation-related pathways, such as EMT and Pan-F TBRS, enriched in cluster 2, suggesting an immune excluded phenotype. The results above also proposed that cluster one was significantly associated with DNA damage control, which usually associated with higher TMB, and implied a more favorable immunotherapy outcome.

### Construction of Ferroptosis Related Genes Score and Validation of the Prognostic Ability

**FIGURE 4:**
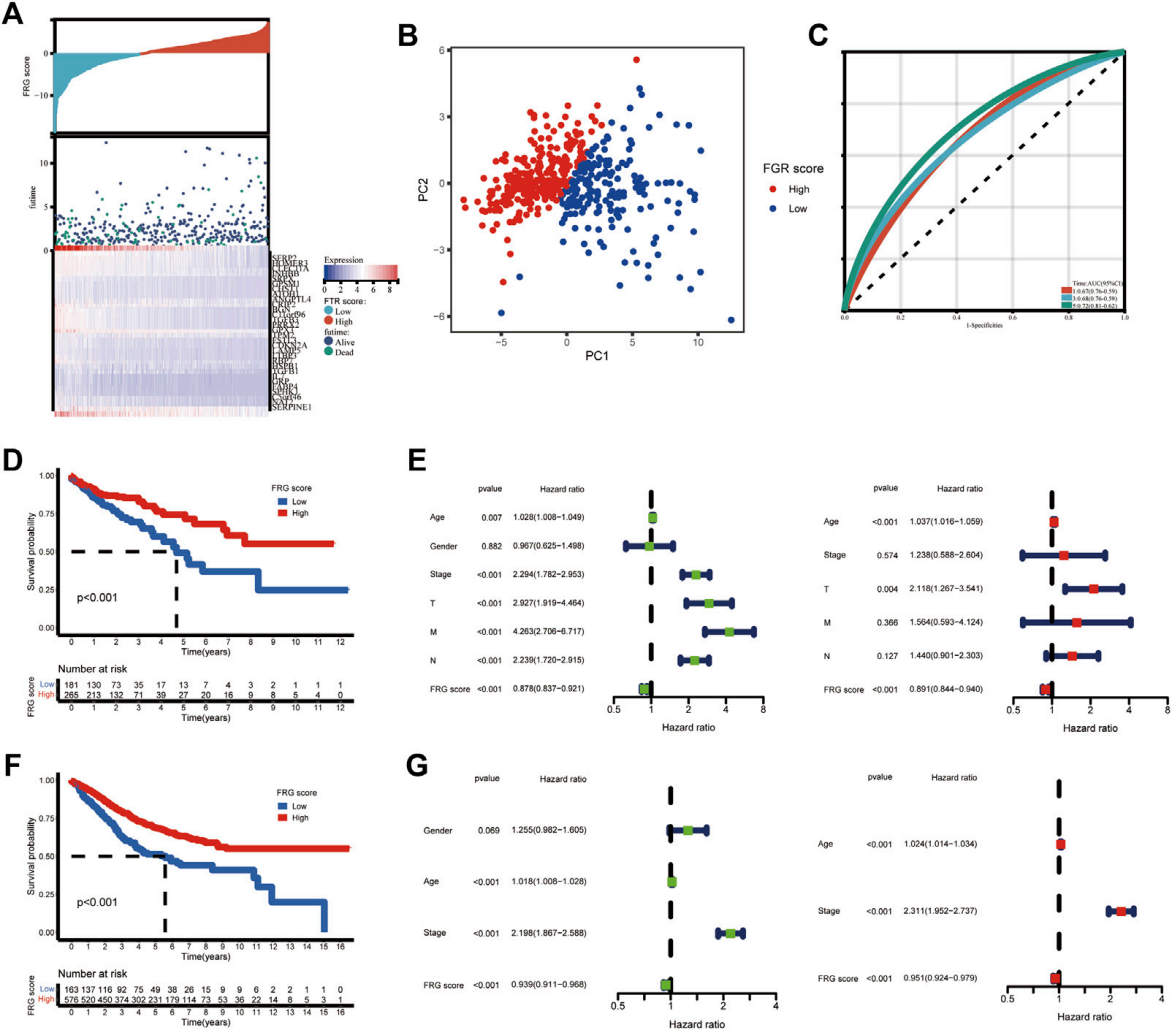
Construction of the FGR score and validation of the prognostic ability. **(A)** Ranked dot and scatter plots showing the FRG score distribution and patient survival status. **(B)** PCA analysis based on the FRG coring system. The high and low risk patients are represented by red and blue dots, respectively. **(C)** ROC curves to predicting the sensitivity and specificity of 1,3, and 5 years survival, according to the FRG score. **(D)** Kaplan-Meier analysis of the OS of patients in TCGA cohort between the two groups. **(E)** Univariate and multivariate Cox regression results revealing the relationship of gender, age, TMN stage and FRG score with prognosis in TCGA cohort. **(F)** Kaplan-Meier analysis of the OS of patients in GEO cohort between the two groups. **(G)** Univariate and multivariate Cox regression results revealing the relationship of age, stage and the FRG score with prognosis in GEO cohort.

Given the unique complexity and heterogeneity of ferroptosis, a scoring system based on these phenotype-related genes was developed to calculate the expression pattern of FRGs in individual COAD patients, which we called the FRG score. First, we used the limma package to identify 586 ferroptosis phenotype-related DEGs (log FC = 0.585, FDR = 0.05). “We next used univariate cox analysis to identify the DEGs that were connected to survival outcome.” 31 of the 586 phenotype-related DEGs show predictive significance (*p* < 0.05). We then used principal component analysis (PCA) to create an FRG grading system based on the 31 DEGs. The FRG score distribution plot demonstrated that as FRG scores grew, so did survival times and rates (Figure 4A). The distinct dimensions between the high and low-FRG score groups were observed through PCA analysis (Figure 4B). The 1, 3, and 5 years FRG score survival rates were represented by AUC values of 0.67, 0.68, and 0.72, respectively (Figure 4C). Patients with high scores had significantly better survival rates than patients with low scores (log-rank test, *p* < 0.001) as per Kaplan–Meier survival curves (Figure 4E).

According to the Univariate findings, a lower FRG score was substantially related to an unsatisfactory OS [hazard ratio (HR): 0.878; 95% confidence interval (CI): 0.837–0.921; *p* < 0.001]. Higher age, advanced stage, deeper invasion, positive lymph nodes, and distant metastases are further clinicopathologic factors linked to poor survival (Figure 4E). In a multivariate analysis, FRG, T stage, and age remained independently linked with overall survival, with an HR of 0.891 (CI: 0.844–0.940, *p* < 0.001) as shown in Figure 4F. We confirmed these findings in GEO cohorts (GSE17536, GSE39582). The survival analysis revealed that the low-risk group had a much better prognosis than the high-risk group (Figure 4G). FRG score remains strongly linked with COAD patient survival time (Figures 4H,I).

### Evaluation of the Difference Among High and Low FRG Score Groups in Tumor Microenvironment

**FIGURE 5:**
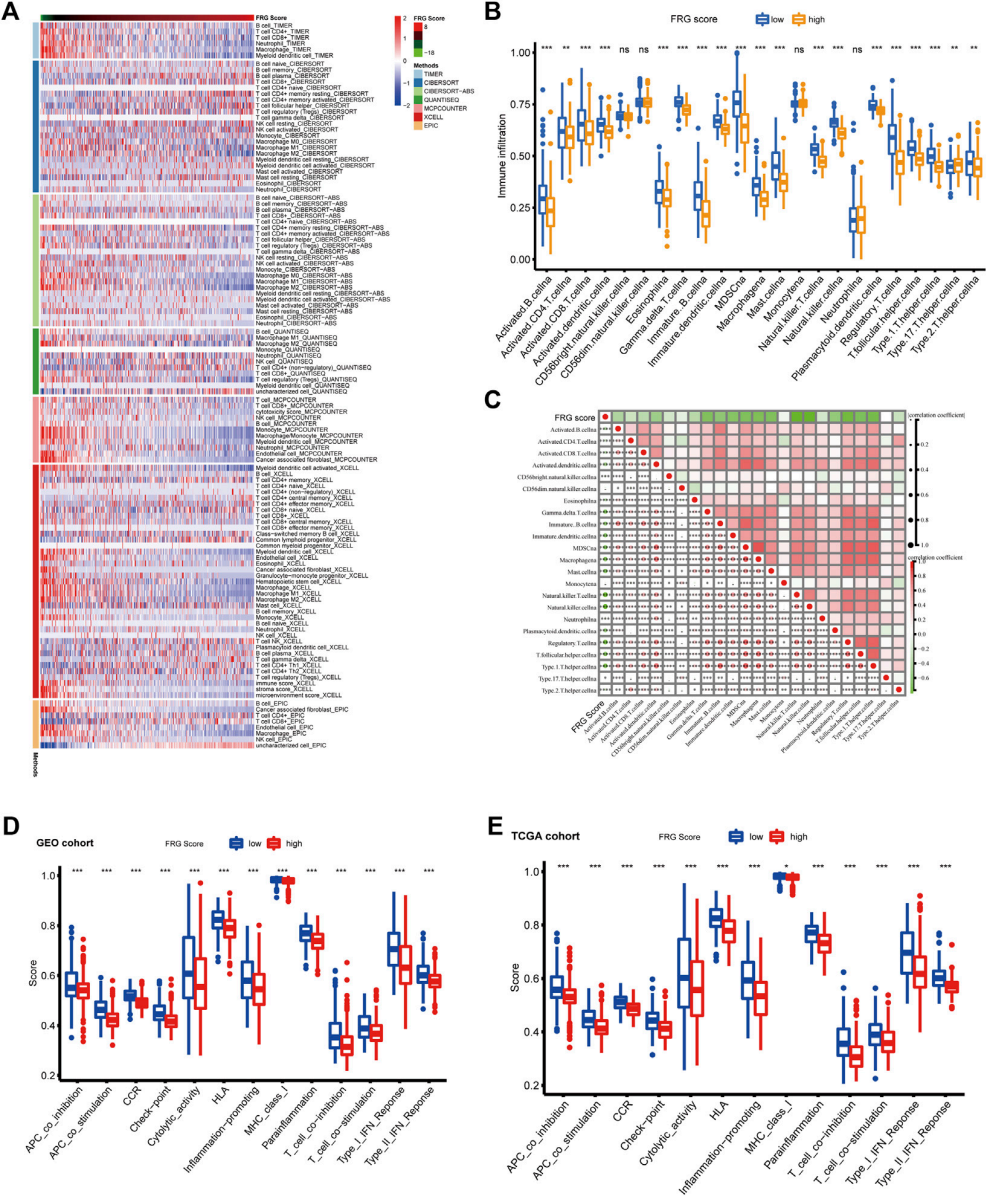
Immune landscape in high and low FRG score groups. **(A)** Heatmap for immune infiltrating cells based on TIMER, CIBERSORT, CIBERSORT-ABS, QUANTISEQ, MCP counter, Xcell and EPIC algorithms among high and low risk group. Blue and red represent low and high infiltrating level, respectively. **(B)** The abundance of each TME infiltrating cell in high and low FRG score groups. The upper and lower ends of the boxes represent interquartile range of values. The lines in the boxes represent median value, and black dots show outliers (*p < 0.05; **p < 0.01; ***p < 0.001). **(C)** Correlation heatmap showing correlations between the FRG score and immune infiltrating cells evaluated by ssGSEA methods. Red and green represent low and high Pearson correlation coefficient, respectively (*p < 0.05; **p < 0.01; ***p < 0.001). d-e. The enrichment score of immune function pathways in high and low FRG score groups. **(D)** GEO cohort **(E)** TCGA cohort. The upper and lower ends of the boxes represent interquartile range of values. The lines in the boxes represent median value, and black dots represent outliers (*p < 0.05; **p < 0.01; ***p < 0.001).

Figure 5A illustrates a heatmap of tumor-infiltrating immune cells through EPIC algorithms, Xcell, MCP counter, TIMER, QUANTISEQ, CIBERSORT, and CIBERSORT-ABS. Based on ssGSEA of TCGA-COAD data, analysis of immune cell subpopulations revealed higher immune infiltration levels in the low-FRG score group (Figure 5B). The correlation heatmap showed a strong correlation between FRG scores with most types of immune infiltrating cells. Differential analysis of immune functions pathways using ssGSEA revealed immune stimulation pathways (INF response, CCR, co-stimulation, inflammation regulation, cytolytic activity, HLA and MHC) and immune inhibition pathways including the checkpoint, co-inhibition was significantly different between the high and low-risk groups. While patients with low FRG scores were prominently related to high level of immune infiltration and immune activation pathways. However, patients with this low FRG score did not show a matching survival advantage. Previous studied suggested tumors with immune-excluded phenotype also showed the presence of abundant immune cells, while these immune cells were retained in the stroma surrounding tumor cell nests rather than penetrate their parenchyma. Thus, we speculated that our FGR score could serve as a predictor of immune-excluded subtypes.

### Development of a Nomogram to Predict Survival

**FIGURE 6:**
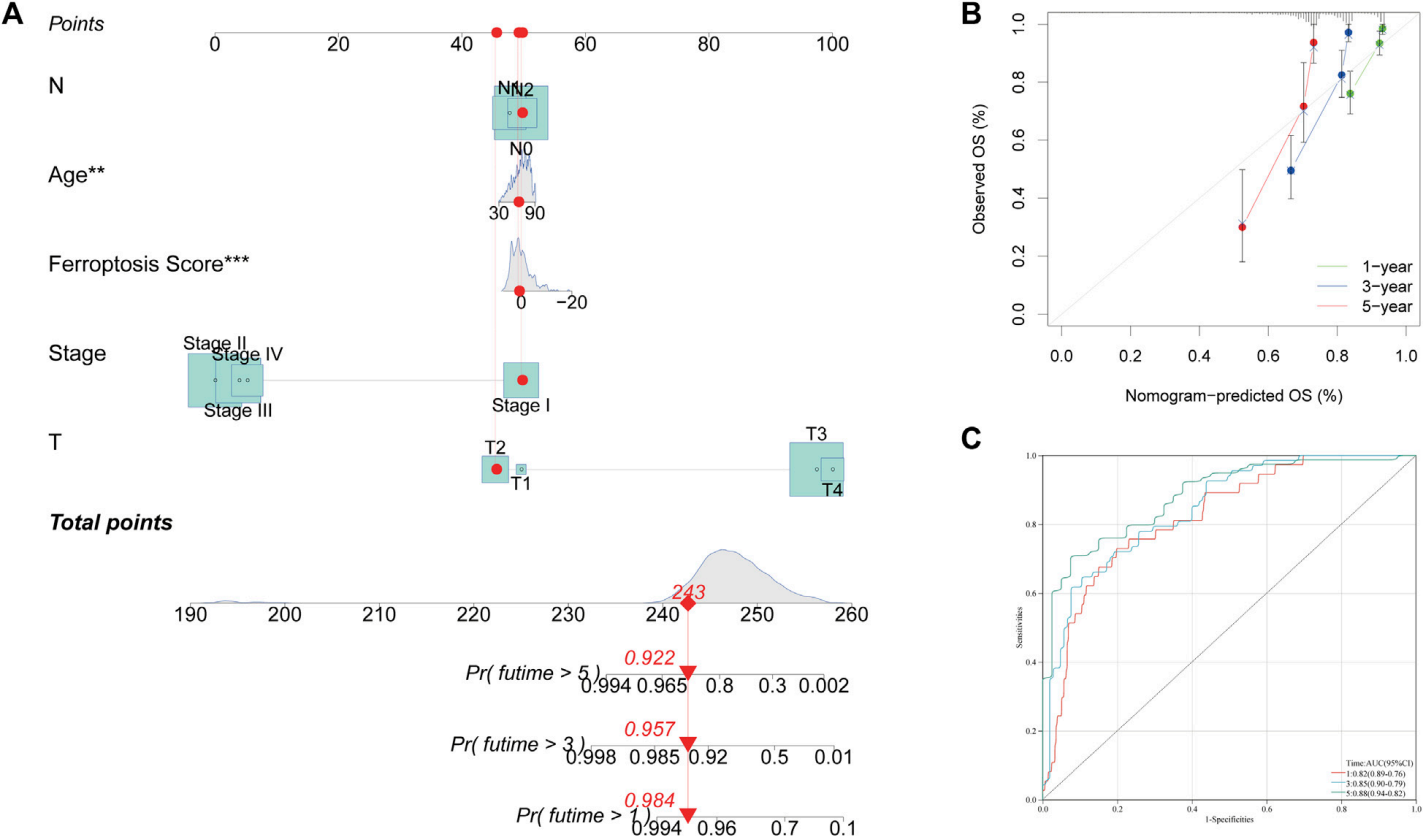
Construction and validation of a nomogram. **(A)** Nomogram for predicting the 1, 3 and 5 years OS of COAD patients. **(B)** Calibration curves of the nomogram for predicting 1, 3 and 5 years OS of COAD patients. **(C)** ROC curves for predicting 1, 3 and 5 years OS of COAD patients.

For predicting the 1, 3, and 5 years OS rates, a nomogram was developed using the FRG score and clinicopathological characteristics (Figure 6A). Age, FRG score, and patient stage were all used as predictors. The AUC trials of our study revealed good precision on the nomogram model for OS at 1, 3, and 5 (0.82, 0.85, and 0.88, respectively) in the TCGA-COAD cohort, indicating that the nomogram possessed a strong prediction ability (Figure 6B). Calibration plots revealed that the proposed nomogram performed similarly to an ideal model (Figure 6C).

## Discussion

Ferroptosis can overcome malignant cells’ resistance to chemotherapy and assist in removing damaged cells. As a result, it could be a novel strategy for treating tumors (Mou et al., 2019) (Ghoochani et al., 2021) (Ye et al., 2020). According to two recent studies, ferroptosis caused by T cells in cancerous cells provided new insights of improving curative effect of the PD-1/PD-L1 antibody, and only a moderate influence of the PD-L1 antibody has been identified in ferroptosis-insensitive tumor cells (Wang, et al., 2019) (Lang et al., 2019). A lack of thorough understanding of the role of several FRGs in TME infiltration is because most research focuses on single TME infiltrating cells or single FRGs. The understanding of the role of ferroptosis in shaping TME, thereby effecting therapy efficiency and prognosis, can be improved by identifying the role of unique transcriptional ferroptosis subtypes in ferroptosis and TME antitumor immunity.

We found two unique forms of ferroptosis based on 51 FRGs that were differentially expressed. They were characterized by distinct biological processes. One of the characteristics of Cluster one was the activation of the DDR pathway. Angiogenesis features, FGFR3-related genes, and pan fibroblast TGF-β response characteristics (Pan-F TBRS) were detected in cluster two based on the TCGA data. According to previous research findings, immune cells were prevalent in tumors with an immune-exclusion pattern. Rather than entering the parenchyma of tumor cells, these immune cells remained in the stroma around them. T-cell suppression was thought to be inhibited by stroma activation in TME (Chen and Mellman, 2017). Stromal activation-related pathways, such as EMT pathways and Pan-F TBRS, enriched in cluster 2, suggested it has an immune excluded phenotype (Tang B. et al., 2020). Thus, we defiend these two subtypes as DNA damage repair and immune-excluded phenotype. The genome’s integrity is dependent on the DNA damage repair system (O'Connor, 2015). DDR pathway-related genes were strongly associated with a higher tumor mutation burden and predicted optimal anti-PD-1/PDL1 immunotherapy efficacy (Teo et al., 2018; Arora et al., 2019). Earlier research has found that gene alterations are linked to immune treatment response (Dong et al., 2017) (George et al., 2017) (Burr et al., 2017). Therefore, we evaluated the variation of TMB and somatic mutations between different ferroptosis subtypes. Consistent with previous studies, there was a significant difference between ferroptosis clusters one and two in COAD. TMB-high tumors are more likely to develop new immunogenic neo-antigens, increasing their immunotherapy susceptibility (Riaz et al., 2016). our study showed that ferroptosis was linked to the stromal and immunological TME as well as the tumor mutation burden. To above reasons, FRGs may play a significant role in the immune response to tumors.

A comprehensive assessment of the ferroptosis subtypes will enhance our understanding of TME cell-infiltrating characterization. Considering the individual heterogeneity of FRG expression, it was urgently demanded to quantify the mRNA transcriptional pattern related to ferroptosis of individual tumor. For that, we established a set of scoring system to evaluate the ferroptosis pattern of individual patients with colon cancer the FRG score. Significant links were found between the FRG score and TME features, and immunological functioning. A lower FRG score was associated with a higher level of infiltration as well as immune system activation and inhibitory pathways. (Liu et al., 2020). A comprehensive investigation of ferroptosis’s aberrations and cancer-related functional consequences was conducted by Liu et al., 2020. The high-FPI group had a greater abundance of IL-6/JAK/STAT3 (immune) signaling pathways and epithelial-mesenchymal transition (stromal). Additional studies showed a link between higher ferroptosis level and poorer survival in adenocarcinoma of the lung, liver hepatitis cell carcinoma (LIHC), kidney renal papillary cell carcinoma (KIRP), kidney renal clear cell carcinoma (KIRC), and GBM, suggesting the dual role of ferroptosis in tumor progression. Therefore, regulating the ferroptosis level in TME by targeting multiple potential targets may help patients improve their prognosis.

In this study, we showed the transcriptional pattern of ferroptosis related genes played a nonnegligible role in shaping different stromal and immune TME landscape, implying ferroptosis could further determine the immune phenotypes of tumors and guide the more effective clinical practice. We also demonstrated that FRG score has a significant correlation with prognosis of colon cancer and could act as an independent prognostic biomarker for predicting patients’ survival. This result has been verified in the large-scale TCGA and GEO cohorts.

However, there are a few limitations that need to be pointed out. The majority of our conclusions are based on information that was collected in the past. Prospective clinical validation in a larger COAD cohort is now required. The significance of ferroptosis in shaping TME characteristics in COAD was unknown, and it should be examined experimentally in future studies.

## Data Availability

Publicly available datasets were analyzed in this study. The data that support the findings of this study are available in TCGA database at (https://tcga-data.nci.nih.gov/tcga/) and GEO database at (https://www.ncbi.nlm.nih.gov/).
